# People’s Responses to Nuclear Weapons: Mapping Post-Cold War Research

**DOI:** 10.1177/17456916251404895

**Published:** 2026-02-26

**Authors:** Astrid Kause, Helen Fischer, Zia Mian, Susan T. Fiske

**Affiliations:** 1 School of Sustainability, Leuphana University Lüneburg; 2 Harding Center for Risk Literacy, Faculty of Health Sciences, University of Potsdam; 3 Leibniz-Institut für Wissensmedien, Tübingen, Germany; 4 Program on Science and Global Security, Princeton University; 5 Department of Psychology and School of Public and International Affairs (emerita), Princeton University

**Keywords:** systematic map, nuclear weapons, existential risk, beliefs, feelings, actions, knowledge

## Abstract

Nuclear weapon threats are increasing and may be comparable to levels not seen since the worst periods of the Cold War. There could be value in psychologists documenting and explaining people’s responses to nuclear weapons. More than 3 decades have passed since the last major reviews of people’s responses to nuclear weapons. We thus aimed to understand how psychologists and researchers from related fields have empirically studied responses to nuclear weapons since the end of the Cold War. We systematically mapped articles reporting on people’s responses. A search in Web of Science and Scopus identified 18,505 hits. Screening resulted in 256 suitable articles. We assessed (a) publication patterns, including how many articles focused on responses to nuclear weapons, when those articles were published, and in which field; (b) the research community, namely author collaborations and focal journals; (c) research themes, as indicated by cocitation networks and theoretical backgrounds; and (d) the validity, generalizability, and replicability of empirical findings, as indicated by adequate samples and validated measures. We found renewed interest in the field but not yet a coherent research community and only some evidence for its evolution from occasional, scattered, one-off studies toward a coherent and coordinated scholarly field.

Nuclear weapons are back in the global public debate. Concern increases about the risk of nuclear weapon use and the renewed commitment to nuclear arsenals, especially involving the countries with the largest nuclear forces. In 2024, *The New York Times* launched “At the Brink,” a series about current nuclear weapons risks, noting the “once again growing threat of nuclear weapons” (Kingsbury, 2024, para. 13).

The problem has grown increasingly severe over more than 2 decades. In 2001, the United States withdrew from the 1972 Anti-Ballistic Missile Treaty (Neilan, 2001). In response, Russia withdrew from the Strategic Arms Reduction Treaty (START) II, suspended the Treaty on Conventional Armed Forces in Europe, developed weapons intended to overcome U.S. ballistic missile defenses, and built systems the United States claimed violated the range limits agreed on in the 1987 Intermediate-Range Nuclear Forces (INF) Treaty (Neuneck, 2019). Attention to the dangers of nuclear weapons was further spurred by U.S. policies under the first Trump administration, which took office in 2017. By 2020, the United States had withdrawn from the INF Treaty and the 2015 Iran nuclear deal, refused to extend the 2010 New START agreement with Russia—which capped the number of their deployed strategic nuclear weapons—and ruled out ratification of the 1996 Comprehensive Nuclear-Test-Ban Treaty (Wittner, 2024). In 2021, evidence emerged that China was building up its nuclear arsenal (Warrick, 2021). In 2022, Russia threatened to use nuclear weapons as part of its war on Ukraine (Mills, 2024; Sanger, 2024). Unsurprisingly, United Nations (UN) Secretary-General António Guterres warned in August 2022 that the world faced “a time of nuclear danger not seen since the height of the cold war” (UN, 2022, para. 4).

Global nuclear arms reduction and disarmament efforts started in the Cold War have slowed down. The five nuclear-armed member states of the Treaty on the Non-Proliferation of Nuclear Weapons (the United States, Russia, the United Kingdom, France, and China) are seen by many of the 186 nonweapon states parties to the treaty to be failing to take concrete disarmament steps despite having agreed to do so in 2000 and 2010, contributing to a “comprehensive regime crisis” of the nuclear order (Baldus et al., 2021, p. 197). As of 2025, these five states are estimated to together hold more than 11,800 nuclear weapons of a global total of about 12,300 weapons, whereas the remaining 500 or so weapons are held by the four other nuclear armed states—Israel, India, Pakistan, and North Korea (Kristensen et al., 2025).

Motivated by the lack of progress toward disarmament and concerned about the global humanitarian impacts of nuclear war for innocent bystander states, societies, and nature, 122 non-nuclear weapon states in 2017 agreed on the Treaty on the Prohibition of Nuclear Weapons (TPNW; Kmentt, 2021). The TPNW, which entered into force in 2021, establishes a set of norms and legal obligations to “never under any circumstances” develop, test, produce, acquire, possess, stockpile, use, or threaten to use nuclear weapons while requiring states with nuclear weapons programs to eliminate them (Kmentt, 2021). It has 95 signatory states (i.e., almost half of the 193 UN member states), and 74 are ratified states parties, all of which have accepted to be bound by the treaty (UN, 2025b). In a recent joint statement, the 95 TPNW signatory states explained their concerns, noting that

heightened geopolitical tensions, further expansion and modernization of nuclear arsenals, the increasing salience of nuclear weapons in military and security doctrines, including through security guarantees and the growing dangers of nuclear proliferation and potentially devasting nuclear arms race demand immediate and decisive action from all of us. (States Parties and Signatory States to the TPNW, 2025, p. 2)

Reflecting this concern, the UN General Assembly recently and overwhelmingly supported a resolution to set up a study of the effects of nuclear war (UN, 2024). This UN study will be the first study on nuclear war since the 1980s (Mian, 2024a, 2024b). Support of the TPNW as well as this resolution may reflect that global concern about nuclear weapons remains high.

## Psychology’s Role

Psychologists have a role to play in documenting and understanding people’s reactions to this existential threat, as do researchers from related fields such as political science (Lifton & Falk, 1982). Insights into public responses to nuclear weapons can also shape nuclear policies (Herzog et al., 2022; Onderco et al., 2023; Rattinger, 1987). Accordingly, psychologists have a long tradition of empirically studying public responses to nuclear weapons (Morawski & Goldstein, 1985). Several literature reviews from psychology and political science (such as S.T. Fiske, 1987; Russett, 1990; Schatz & S.T. Fiske, 1992) have synthesized research on public responses to nuclear weapons. Psychologists have also called for taking social responsibility, including through research on (Morawski & Goldstein, 1985; Vigne et al., 1988) and teaching about (Murphy & Polyson, 1991) nuclear weapons, and thus contributing to the welfare of humanity. In addition, in 1982, American Psychological Association members widely supported a resolution calling for an immediate halt to the nuclear arms race (Abeles, 1983; Polyson et al., 1988).

We take stock of what psychologists and researchers from related fields have been doing since the end of the Cold War, almost 40 years since the last reviews by psychologists of people’s responses to nuclear weapons (S.T. Fiske, 1987; T.W. Graham, 1988; Schatz & S.T. Fiske, 1992). Our analytical lens captures practical aspects of the field’s development, such as publication patterns, including how many articles focused on responses to nuclear weapons, when those were published, and in which field. We also examine author collaborations and focal journals. Our thematic lens captures research themes, as indicated through cocitation networks and theoretical backgrounds mentioned in articles. Our methodological lens captures the validity, generalizability, and replicability of empirical findings, as indicated by adequate samples and validated measures. Each of these metrics shows that research on public responses to nuclear weapons has not yet fully developed into a mature science.

## Publication Patterns: Quantity over time, in different fields

Article quantity reflects the dynamics within a research field. Studies on the development and evolution of science suggest that findings viewed as sufficiently innovative to be a “discovery” by a small research group can inspire others (Kuhn, 1970) who pick up these discoveries, develop new research practices, and share these, for better understanding initial discoveries and their applications (Bettencourt & Kaur, 2011). Research on nuclear weapons evolving in psychology and related fields may thus follow a growth trajectory, as does research on other global challenges such as climate change (e.g., Berrang-Ford et al., 2021).

Article quantity over time may also reflect policy change (Bettencourt & Kaur, 2011). Nuclear threat has ebbed and flowed since the number of nuclear warheads decreased rapidly toward the end of the Cold War (Kristensen et al., 2024). Although the perceived risk from nuclear weapons remained lower throughout the following decades, it substantially increased again, particularly after the onset of the Russian–Ukrainian war (Bulletin of the Atomic Scientists, n.d.). Other political events (e.g., election of leaders who take aggressive stands in states with nuclear weapons or states that may seek such weapons) likely also link to article quantity.

## Research Community: Author Collaborations and Focal Journals

Whether and how much authors collaborate can convey a field’s developing research community (Bettencourt & Kaur, 2011). Coauthorship networks reflect patterns of author collaborations (Savič et al., 2018). The number of publications per author indicates whether and how much this author contributes to progress within a field (Engler et al., 2024; Savič et al., 2018): “Brokers” publish many articles while collaborating with many others; “solitary” authors publish many articles but collaborate with few others (Engler et al., 2024). A tighter research community would be reflected in many coauthorships.

Another indicator for research-community development is how articles distribute across many or few journals (Engler et al., 2024). Journal variety may reflect whether authors address related questions, draw on similar theories, agree on similar methods, and share existing knowledge for explaining their data.

## Developing a Science: Themes and Theories

A developing research field may come with semantic subclusters of specific themes (Zhang et al., 2021). One indicator for how much authors pick up themes, theories, or methods is whether and how often authors cocite the same or different sets of references (Small, 1973; Trujillo & Long, 2018). This reflects both whether scholarship has received attention across fields as well as key communities within a field (Trujillo & Long, 2018).

Moreover, the theoretical backgrounds mentioned in articles may reflect to what extent research contributes to psychology as a cumulative science overall (Gigerenzer, 2010; Mischel, 2008) and here, to understanding public responses to nuclear weapons as a cumulatively developing subfield. Drawing on the same theories may help overcome the “toothbrush problem” (Mischel, 2008) of siloed research traditions that explain the same phenomena but with different theories, methods, and labels. In contrast to psychology, fields such as biology or physics draw on overarching theoretical frameworks, such as evolutionary theory or relativity theory (Borsboom et al., 2021). Psychology, and possibly also research on public responses to nuclear weapons, however, may be characterized by smaller, theory-rich bubbles (Borsboom et al., 2021).

## Generalizable and Replicable Findings: Samples and Validated Measures

The generalizability and replicability of empirical research in psychology rises (and falls) with sample size, quality, and type (Vazire et al., 2022). Psychologists have aimed at improving statistical power by conducting power analysis (Collins & Watt, 2021); by using new methods, such as multisample comparisons (Klein et al., 2018; Vlasceanu et al., 2024); and by aiming for higher transparency and openness, for example, by using preregistrations (Krypotos et al., 2022). Research on public responses to nuclear weapons may similarly reflect these trends.

Behavioral scientists generally (Henrich et al., 2010; Renkewitz & Heene, 2019; Zickar & Keith, 2023) as well as psychologists studying public responses to nuclear weapons specifically (S.T. Fiske, 1987) have called for using representative rather than convenience samples. Nuclear threat occurs globally but varies in local impacts. Studies on public responses to nuclear weapons may thus draw on different populations and include people from nuclear-armed states, their allies, or nonarmed states. They may also focus on particularly vulnerable subgroups, such as nuclear weapon production workers, victims of testing, or children. Overall, a given sample should represent its explicitly intended population.

Moreover, the development and use of shared methods and measures potentially increases research validity. Qualitative and mixed-methods studies (S.T. Fiske, 1987; Schoonenboom & Johnson, 2017) allow first assessing the overall range of responses or generating hypotheses (Creamer & Reeping, 2020). Quantitative follow-up surveys can then test hypotheses, using large samples (Bruine de Bruin & Bostrom, 2013).

Validity can improve through the development and use of validated measures for precisely defined responses, rather than ad hoc measures (Vazire et al., 2022). Validated measures enable replication and research progress (Mischel, 2008) and can be used in studies on people’s responses to nuclear weapons (Mayton, 1988).

Validated measures could address different psychological responses to nuclear weapons. Responses include people’s *beliefs*. Beliefs are subjective assumptions about an object or state of the world. They are based on limited information a person may have (Wirtz, 2017). Here, beliefs may include the perceived likelihood of nuclear war or nuclear war impacts (S.T. Fiske, 1987; S.T. Fiske et al., 1983). People may also form beliefs about the impact and effectiveness of nuclear policies. Responses may also include *feelings*. These are people’s mental state of worry, that is, subjective “distress or agitation” in anticipation of an event (American Psychological Association, n.d.), such as nuclear war. Feelings also include emotions, such as sadness (S.T. Fiske, 1987; Schatz & S.T. Fiske, 1992), as well as “nuclear anxiety” (Newcomb, 1989) and associated visceral reactions (Wirtz, 2017). Responses also include *actions*, that is overt behaviors (American Psychological Association, 2018), such as writing to elected representatives or donating to a nongovernmental organization for addressing issues such as a nuclear freeze (Schatz & S.T. Fiske, 1992) or nuclear disarmament (Russett, 1990). Last, *knowledge* reflects actual cognitive representations of objects (Wirtz, 2017), or, here, understanding of and familiarity with (American Psychological Association, n.d.) nuclear weapons and nuclear policies (T.W. Graham, 1988). Correct knowledge enables people to make informed decisions (Bruine de Bruin & Bostrom, 2013) about nuclear issues and, generally, hold policymakers accountable (Fialho, 2021).

## Research Questions

We examined whether and how research on public responses to nuclear weapons has evolved since a local peak of activity during the Cold War (e.g., S.T. Fiske, 1987). Specifically, we focused on (1) publication patterns, namely how many articles were published, when, and in which field; (2) research community, namely how authors collaborated and where their articles were published; (3) the research themes and theoretical backgrounds; and (4) whether adequate samples and validated measures differentiated public responses to nuclear weapons.

## Method

Established formal systematic review guidelines for empirical research (James et al., 2016) from psychology (Siddaway et al., 2019) and the environmental sciences (Pullin et al., 2022) informed this systematic map.

### Search and screening

For validating the overall search strategy, we collected recommendations for empirical articles on responses to nuclear weapons from experts who had recently published on this topic. For designing a search string, we adopted terms from their titles and abstracts, terms from S.T. Fiske (1987) and terms from articles that cited S.T. Fiske (1987; see Table S1 in the Supplemental Material available online).

Scopus and Web of Science were searched between January 23 and 30, 2024. The number and type of hits differed between two different versions of the Scopus website that were accessible at the time. Search results from both Scopus versions were therefore included here. This resulted in a total of 18,505 hits, after removing duplicates (Fig. S1). We manually added articles that cited S.T. Fiske (1987) according to Google Scholar on April 4, 2024. We also added 19 articles that were not found through databases because databases did not include the journal (e.g., Boehnke & Schwartz, 1997; Fialho, 2021) or omitted the abstract (e.g., Newcomb et al., 1992; Sagan & Valentino, 2017; Van Hoorn & French, 1988). We searched again but with a shorter substring that included the terms “freeze OR race* or *arm*” on April 15, 2024 (Table S1). This was because some identified articles used the term “[arms] race” (Plous, 1993), “freeze,” or “disarmament,” which were not included in the original search strings.

**Fig. 1.**
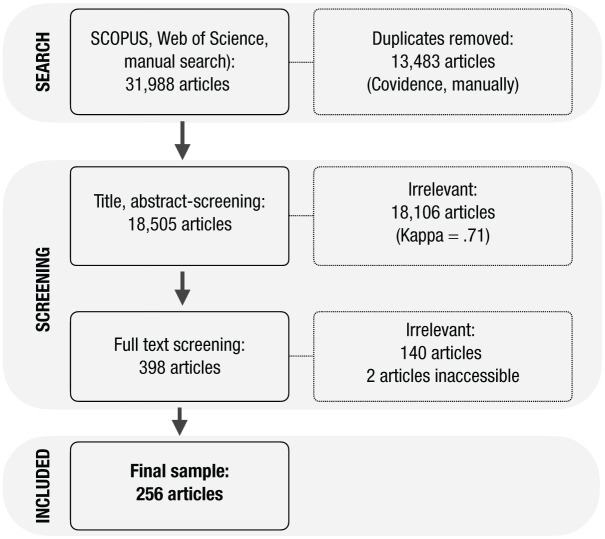
Search and screening procedure. The search and screening procedure followed established guidelines for systematic review protocols (Pullin et al., 2022; Siddaway et al., 2019). Details are provided in the ROSES systematic map protocol available at https://osf.io/jz5xc. ROSES = Reporting Standards for Systematic Evidence Syntheses.

Articles were screened in two stages (Fig. 1). First, screening criteria were developed using an article subset. Screening criteria were applied by A. Kause and H. Fischer to titles and abstracts. Because of an initial insufficient interrater reliability (Cohen’s κ = .52; *n* = 180; Altman, 1991), criteria were revised (Table S2). Interrater reliability subsequently increased to .71 (95% proportionate agreement) for approximately 10% of the articles (*n* = 1,745). The remaining articles were then screened by A. Kause.

Second, A. Kause screened full texts. The same set of screening criteria was applied but with two additional restrictions: Articles were excluded if they addressed clinical mental health more generally, such as depression, cognitive dysfunction, or other mental disorders, or if they described responses to or the preparedness of health professionals for nuclear, radiological, and chemical hazards more generally (Table S2). We did this because (a) benchmark articles, as well as earlier reviews (S.T. Fiske, 1987; Schatz & S.T. Fiske, 1992), did not include clinical mental-health impacts either; (b) articles addressing mental health often report on specific different methods, such as epidemiological studies that may require a different type of review or meta-analysis; and (c) our initial search strings did not include a complete list of relevant terms describing the range of possible clinical mental-health impacts, such as posttraumatic stress disorder, depression, stress, or trauma. In the Discussion section, we thus suggest ways forward for a separate review with a focus on clinical mental health. For two articles the full text could not be retrieved (Garatti & Rudnitski, 2007; Ayvaci & Bakirci, 2012).

### Data extraction and analysis

Using a mixed-methods approach, we analyzed article metadata retrieved from scientific databases (e.g., Engler et al., 2024). We also manually extracted information from articles. To this end, A. Kause developed a manual coding scheme (Table S3). The coding scheme was iteratively refined, together with H. Fischer, until sufficient agreement was reached.

For examining publication patterns (how many articles, published when, and in which field; Research Question 1), we traced publication dates. We also extracted the first author’s institutional affiliation and coded their disciplinary field accordingly (Table S3).

For examining how the research community had evolved through author collaborations and focal journals (Research Question 2), we analyzed coauthor network density and the number of publications per author (Engler et al., 2024). Density reflects the degree of collaboration between authors. Density was calculated by taking the number of actual coauthorships and dividing that number by the number of theoretically possible coauthorships between all authors (Engler et al., 2024). A coauthor network reflects whether authors are brokers involved in multiple collaborations or solitary authors with large numbers of publications but few collaborations (Engler et al., 2024). We also extracted the range of journals where articles were published and the number of articles published per journal (Table S4).

For examining themes and theories (Research Question 3), we first conducted a cocitation analysis (Small, 1973). Because software did not allow us to conduct cocitation analyses on merged metadata files from Scopus and Web of Science (see https://www.bibliometrix.org/home/index.php/faq), we downloaded available article metadata from Web of Science. This was because the quality of Web of Science metadata is higher, compared to Scopus. An overview of included (*n* = 211) and missing (*n* = 45) articles is available at https://osf.io/jz5xc. We analyzed cocitations in this Web of Science metadataset, using the R package bibliometrix (Aria & Cuccurullo, 2017).

For examining conceptual frameworks, we extracted any theoretical backgrounds mentioned in article introductions. We did not extract post hoc theoretical explanations mentioned only in article discussion sections or descriptions of mere empirical links between variables (Borsboom et al., 2021). We qualitatively analyzed theoretical backgrounds as follows. We coded broad frameworks or paradigms (Partelow, 2023), such as rationality or bounded rationality, or deterrence theory. We differentiated those from more precise theories (Borsboom et al., 2021). Examples for theories are protection motivation theory (Rogers, 1975), moral foundations (J. Graham et al., 2013), or the psychometric paradigm (Slovic et al., 1984). We also coded more fine-grained psychological phenomena (Borsboom et al., 2021), such as precise causal mechanisms or hypotheses. Examples are psychic numbing (Lifton, 1991), heuristics (Peters et al., 2004), or the cushion hypothesis (Hsee & Weber, 1999). Last, we coded policy norms that may explain responses, as often examined in empirical studies from political science. These norms may be the nuclear taboo (Tannenwald, 2007), virtuous violence (A.T. Fiske & Rai, 2014), or rallying around the flag in times of crises (Lamare, 1987). Here, we qualitatively describe the range of theoretical backgrounds that occurred more than twice (Tables S5a–S5d). Order reflects frequency of occurrence across articles. The full range of theoretical backgrounds extracted is provided at https://osf.io/jz5xc.

For examining samples and measures (Research Question 4), we coded whether articles reported on representative or convenience samples from the general population; on students; on more specific subgroups such as children, adolescents, older adults, and experts; or on activists. We extracted overall sample sizes reported in articles.

For measures, we coded whether articles focused on beliefs, feelings, actions, or knowledge. We extracted references authors cited for the validated scales they reported using for measuring each of those response types. We then examined how specific scales addressed each response type (Table S6 and Supplementary Information S7).

### Data availability and transparency

We uploaded files linked to search, selection, and extraction to https://osf.io/jz5xc. These include articles identified in Scopus and Web of Science and articles retained after full text screening for data extraction, the Reporting Standards for Systematic Evidence Syntheses systematic map protocol (James et al., 2016), whether an article was included into the cocitation analysis, the cocitation analysis code, and coding of theoretical backgrounds.

## Results

### Publication patterns: Article quantity over time, in different fields

**Fig. 2.**
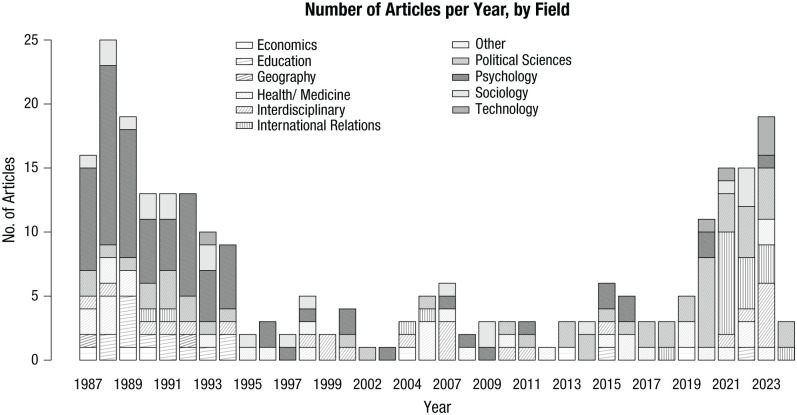
Number of articles published per year since 1987 by author field. Gray shades represent different authors’ fields. Here, “political science” also includes authors from “international relations.” For a color version of this figure, see Figure S2 in the Supplemental Material.

A total of 256 articles were published between 1987 and January 2024. Most articles appeared either around the end of the Cold War, or between 2020 and 2024, with fewer in between (Fig. 2). First authors were often psychologists (*n* = 76; 30%) or political scientists (*n* = 50; 20%). Some articles were published by interdisciplinary authors (*n* = 24; 9%) or authors from international relations (*n* = 21; 8%), sociology (*n* = 21; 8%), or health and medicine (*n* = 18; 6%; Figs. 1 and S2). Articles published between 1987 and 1995 often originated from psychology, education, and health/medicine. In more recent years, articles were published mostly by authors from political science and international relations (Fig. 2; we could not retrieve the field of one first author: Lewis et al., 1989). Most authors worked in the United States or the United Kingdom (Fig. S3a). Fewer came from countries without nuclear weapons or countries affected by nuclear weapons tests. Overall, the U-shaped pattern of publications may reflect sociopolitical events, but with a publication delay, rather than accumulated studies building a scientific foundation.

### Author collaborations and focal journals

**Fig. 3.**
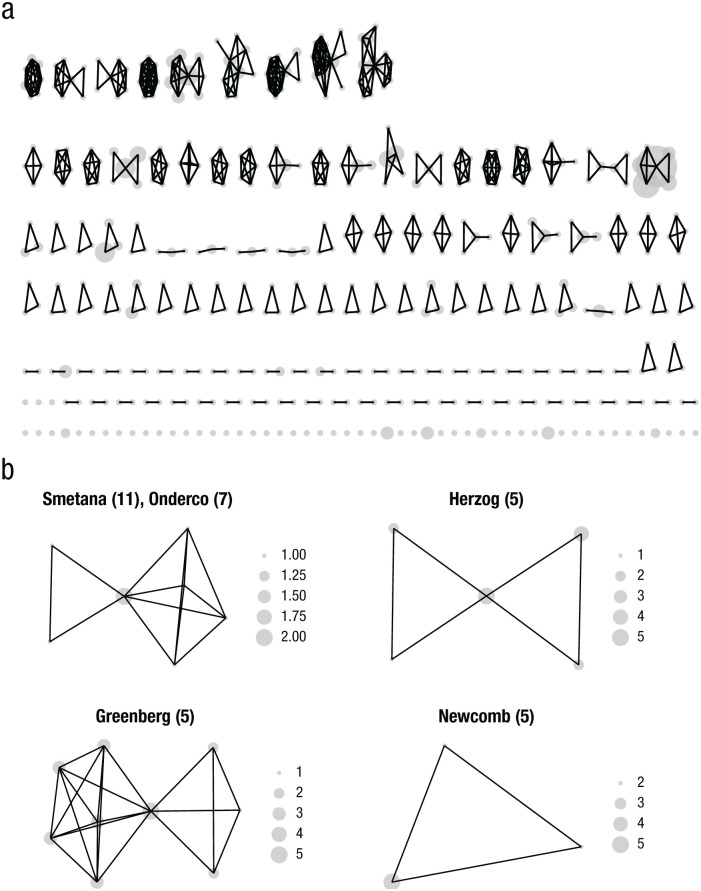
Coauthor networks. Coauthor networks are shown for (a) all authors and (b) only the authors who published more than four articles. Gray nodes represent single authors. Node size reflects the number of articles per author. Lines represent coauthorships. Numbers in parentheses show the number of articles per author.

On average, 483 authors each published 1.28 (*SD* = 0.83) articles. The article distribution per author was highly skewed (Fig. 3a); 404 (84%) authors published only one article, 48 (10%) published two articles, 19 (4%) published three articles, and 12 (3%) published more than three articles. Network density was low (.005): Authors collaborated with, on average, 2.47 others. The collaboration distribution was skewed, with many authors collaborating with, at most, two other authors (Fig. 3a). Only a few authors were involved in multiple collaborations (Figs. 3a and 3b). Figure 3b shows subclusters of authors who published more than four articles. Overall, the research community was scattered.

Articles appeared in 145 different peer-reviewed scientific journals. Table 1 shows the most popular journals that published more than three articles. Otherwise, 10 journals published three articles, 25 journals two articles, and 95 journals just one article (see Table S4). As with the individual authors, journals also mostly published just a few relevant articles.

**Table 1.** Most Popular Journals That Published More Than Three Articles

Journal	Number of articles	SJR	JIF
*Journal of Applied Social Psychology*	13	1.018 (Q2)	2.2
*Journal of Peace Research*	9	1.941	3.4
*Journal of Conflict Resolution*	7	2.138	2.2
*Risk Analysis*	6	0.869	3
*The Journal of Social Psychology*	5	0.945 (Q2)	1.8
*British Journal of Social Psychology*	5	1.665	3.2
*American Journal of Orthopsychiatry*	4	1.068	2.3
*International Studies Quarterly*	4	1.518	2.4
*Journal of Adolescence*	4	1.506	3
*Journal of Global Security Studies*	4	1.062	1.7
*Medicine, Conflict and Survival* (originally published as *Medicine and War*)	4	0.371 (Q3)	NA
*Peace and Conflict: Journal of Peace Psychology*	4	0.465 (Q2)	0.9
*Political Psychology*	4	2.047	4
*Public Opinion Quarterly*	4	1.721	2.9
*Psychological Reports*	4	0.763 (Q2)	1.7

Note: SJRs and JIFs are for 2024 Q1 unless otherwise indicated. Journal information was retrieved from https://www.scimagojr.com/journalrank.php and https://mjl.clarivate.com/home. SJR = SciMago journal rank; JIF = journal impact factor; Q1 = Quarter 1; Q2 = Quarter 2; Quarter 3; NA = not available.

### Themes and theories

**Fig. 4.**
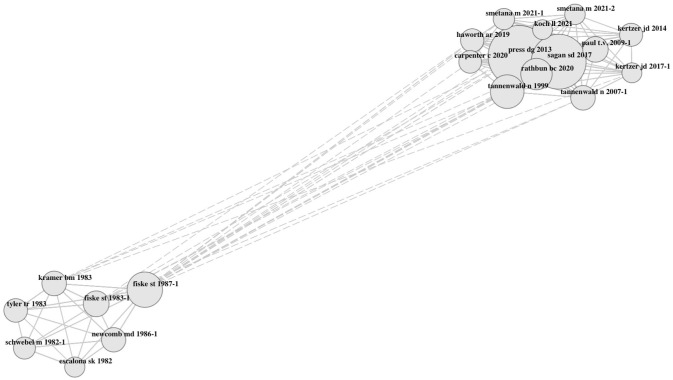
Co-occurrence of the 20 most frequently cocited references. The circle sizes and lines represent how often a reference was cited and whether two references were cited together, respectively. The pattern remained similar when more than 20 references were included.

References clustered according to two themes (Fig. 4). One cluster included references from articles mostly published toward the end of the Cold War that addressed concerns about nuclear war (Goldenring & Doctor, 1986), often in children and teenagers (Escalona, 1982; Schwebel, 1982). This cluster also included articles on attitudes toward nuclear weapons (Kramer et al., 1983), nuclear anxiety (Newcomb, 1991), and psychic numbing (Lifton, 1991). The second cluster included references from articles that were mostly published in the last 12 years. Those addressed public responses regarding international relations (Lin-Greenberg, 2021; Rublee, 2009), such as nuclear policy support (Smetana et al., 2021) or approval of nuclear weapon use in conflicts (Press et al., 2013; Sagan & Valentino, 2017; Smetana & Vranka, 2021). This research often examined how approval can be explained through policy norms such as the nuclear taboo (Tannenwald, 2007) or moral foundations (J. Graham et al., 2013; Kertzer et al., 2014).

Authors drew on a broad range of theoretical backgrounds. We categorized those backgrounds into broader frameworks, more precise theories, specific (causal) mechanisms, or (policy) norms for explaining responses to nuclear weapons (Table 2; for the full range of theoretical backgrounds extracted from articles, see https://osf.io/jz5xc). We qualitatively describe each of those categories in the following paragraphs and provide examples that occurred more than twice. All articles that included each of those categories are listed in Tables S5a through S5d. The order in which the categories are described below reflects the decreasing frequency of occurrence across articles.

**Table 2.** Theoretical Backgrounds Explicitly Mentioned in More Than Two Article Introductions

Category	Theoretical background
Frameworks/ paradigms	Deterrence theory, rationality/rational choice, authoritarianism, social movement literature, social amplification of risk framework
Theories	Social identity theory and social norms, values, moral foundations, stress and coping theory, psychometric paradigm, stress and coping theory, terror management theory, theory of planned behavior, theory of reasoned action, protection motivation theory, nine nuclear orientations
Mechanisms (causal); norms	Gender gap, elite cues/elite public gap, numbing, heuristics, locus of control, generation gap, NIMBY, nuclear taboo, hawks/doves, retributive justice/retaliation, security (prominence), nuclear freeze, rally around the flag, civilian/noncombatant immunity norm

Note: Article references for theoretical backgrounds are listed in Tables S5a through S5d in the Supplemental Material. The full range of theories extracted is provided at https://osf.io/jz5xc. NIMBY = “not in my backyard.”

Broader frameworks (Tables 2 and S5a) mentioned were deterrence theory for explaining approval of nuclear weapon use (e.g., Avey, 2021; Smetana et al., 2022) or activists’ strategies (Sussman & Steel, 1991). Others were rationality or rational choice, including game theory for modeling, for example, arms races (Kramer, 1989; Plous, 1987, 1993); for understanding public opinion about nuclear weapons (Clary et al., 2021); or for predicting financial savings in the face of nuclear war (Russett et al., 1994). Moreover, authors used ideologies, such as authoritarianism, for explaining, for example, war attitudes (Doty et al., 1997), approval of nuclear weapon use (Rathbun & Stein, 2020), or nuclear war concern (van Uzendoorn, 1990). Several authors studied responses through the lens of social interaction. For example, they explained activism by referring to the social movement literature (Edwards & Oskamp, 1992; Marullo, 1988; Tygart, 1987). The social amplification of risk framework was used for understanding responses to nuclear weapon production sites (Flynn et al., 1998).

Authors also drew on more precise theories (Tables 2 and S5b). These included social identity theory or social norms for explaining frames used in nuclear disarmament communications (e.g., Benford, 1993a, 1993b), policy support (Cheng et al., 2023; Fair et al., 2013; Herzog & Baron, 2017; Machida, 2018), responses to nuclear threat (Der-Karabetian, 1992), and nuclear disarmament support (Rigby et al., 1990). Psychological values were used to understand, for example, concern (Mayton 1992; Mayton & Sangster, 1992) or fear of nuclear war (Boehnke & Schwartz, 1997), nuclear weapon acquisition (Dehghani et al., 2010), attitudes (Kristiansen & Matheson, 1990; Ripberger et al., 2011), or activism (Horvath, 1996a, 1996b; Mayton & Furnham, 1994; Sussman & Steel, 1991). Several studies referred to moral foundations or moral values for predicting approval of nuclear weapon use (Dill et al., 2022; Horschig, 2022; Koch & Wells, 2021; Pomeroy & Rathbun, 2024; Rathbun & Stein, 2020; Smetana & Vranka, 2021). One older study related morality to nuclear war concern (van Uzendoorn, 1990). Lazarus’s stress and coping theory predicted feelings (Boehnke & Schwartz, 1997; Boehnke & Wong, 2011; Hamilton et al., 1987, 1989) and activism (Horvath, 1996a, 1996b; McKenzie-Mohr & Dyal, 1991; McKenzie-Mohr et al., 1992) in response to nuclear weapons. The psychometric paradigm was used for examining perceptions of risk from nuclear weapons, risk of war (Bacci et al., 2023; Cutter et al., 1992; Xie et al., 2003), radiation from testing (Peters et al., 2004), and risks related to nuclear weapon production and waste (Flynn et al., 1998; Greenberg, Lowrie, Burger, et al., 2007a; Greenberg, Lowrie, & Mayer, 2007). Risk perceptions and support of nuclear weapon use were also studied through the lens of terror management theory (e.g., Hirschberger et al., 2009, 2015; Horschig, 2022). Ajzen’s theory of planned behavior and his theory of reasoned action (Fox-Cardamone et al., 2000; Halford et al., 1988; Horvath, 1996a; McClenney & Neiss, 1989) or protection motivation theory (Axelrod & Newton, 1991; Horvath, 1996a; McKenzie-Mohr & Dyal, 1991) were used for predicting nuclear activism. Few studies drew on Hamilton’s nine nuclear orientations for predicting concern about nuclear war (Hamilton et al., 1987, 1989; Russo & Lyon, 1990).

Authors drew on more specific (causal) mechanisms for explaining responses to nuclear weapons (Tables 2 and S5c). They explained people’s differing views on nuclear policies with a gender gap (Clements & Thompson, 2022; Lamare 1989) or with differences between political elites and the general public (Smetana & Onderco, 2022). Others referred to Lifton’s (1991) concept of psychic numbing for explaining peoples’ insensitivity to deaths caused by nuclear weapon use (Kaplan, 1988; Slovic et al., 2020). Various responses were further examined through heuristics, such as availability (Chibnall & Wiener, 1988), anchoring (Plous, 1989), or affect (Peters et al., 2004). Moreover, authors studied the role of locus of control for nuclear security perceptions (Herron & Jenkins-Smith, 2002), nuclear war threat (Rudoy et al., 1987; Stewart, 1988), or policy decisions (Rounds & Erdahl, 1988). For understanding responses to nuclear weapon production, some referred to not-in-my-backyard effects (Greenberg, Lowrie, Burger, et al., 2007b; Halfacre et al., 2005). Last, differing views of nuclear policies were explained with a generation gap (e.g., Rattinger, 1987).

Lastly, authors studied whether public opinion reflected specific policy norms (Tables 2 and S5d). For understanding approval of nuclear weapon use or support of nuclear policies, they drew on the nuclear taboo (Allison et al., 2022; Dill et al., 2022); the idea of hawkish or dovish motives (e.g., Haworth et al., 2019; Russett, 1990); retribution, retributive justice, retaliation (Koch, 2024); or security (prominence; Egeland & Pelopidas, 2021; Slovic et al., 2020). Other authors examined whether people would gather behind leaders or governments in times of crises and approve their decisions about nuclear weapons—an effect known as rallying around the flag (Clary et al., 2021; Lamare, 1987). Other prominently studied norms were the noncombatant immunity norm (e.g., Carpenter & Montgomery, 2020; Dill et al., 2022) and support of a nuclear policy freeze (e.g., Chibnall & Wiener, 1988; Hogan & Smith, 1991).

### Samples and measures

Overall, articles included responses by 643,803 participants in 309 studies. Sample sizes reported per article ranged from seven in semistructured interviews with veterans working in nuclear weapons facilities (Murphy et al., 1990) to 284,012 in a quantitative analysis of U.S. votes for nuclear freeze referenda (Cutter et al., 1987). Sample sizes increased over time (Fig. S1). Sample types ranged widely. Few articles (*n* = 57; 22%) reported studies that were representative of the general population. Others examined convenience samples of adults (*n* = 65; 25%) or student samples from psychology (*n* = 26; 10%) or other fields (*n* = 44; 17%). Some articles examined children (*n* = 14; 6%), adolescents (*n* = 39; 15%), or older people (*n* = 3; 1%). Few articles examined diverse experts (*n* = 8; 3%), including U.S. military policymakers (e.g., Pauly, 2018), members of parliament (e.g., Plous, 1993), and workers from weapon production sites (e.g., Mix et al., 2009). Studies on activists were rare (*n* = 9; 4%).

Participant sample locations were mostly in the United States, followed by the United Kingdom, Australia, Canada, and Germany. Some samples were recruited in Asian and European countries (Fig. S3b). Only few articles reported on samples from non-Westernized countries such as Russia (Gulevich & Osin, 2023; Smetana & Onderco, 2023), China (Egel & Hines, 2021), Morocco (Buehler & Banerjee, 2022), or Brazil (Spektor et al., 2022).

Only four studies (2%) were preregistered. Overall, these patterns may indicate some improvement over time regarding statistical power and, therefore, validity.

Some articles reported using validated scales for measuring beliefs, feelings, actions (S.T. Fiske, 1987), and knowledge (Fialho, 2021; Table S5) rather than ad hoc constructed measures (e.g., Russo & Lyon, 1990) or measures from previous studies in which it was unclear whether those measures had been validated (e.g., Gunn & Horvath, 1987; see also Jennings & Lawrence, 1986). Here, we describe how each response type was measured. Because of the substantial variability in ad hoc measures, we refer to validated scales only (for an overview, see Table S6). Table 3 shows some example items for each response type.

**Table 3.** Exemplary Validated Measurement Items for Different Response Types

Response type	Example item	Source
Beliefs	*Likelihood.* What is the probability of a full-scale nuclear war within 10 years? [0 = *0% chance*, 10 = *100% chance*]	Hamilton et al. (1987)
	*Impact.* A nuclear war between the United States and the Soviet Union would cause eventual death for most of our citizens and destroy our economic and political systems. [*strongly agree*, *agree*, *slightly agree*, *slightly disagree*, *disagree*, *strongly disagree*]	Nelson & Slem (1984)
	*Policy beliefs.* Nuclear war can be prevented through building and maintaining a strong nuclear arsenal. As a result of the balance of power and the recognition that there will be no winners in a nuclear exchange, maintaining our nuclear strength will continue to provide effective deterrents to global conflict. [*strongly disagree*, *slightly disagree*, *neither agree nor disagree*, *slightly agree*, *strongly agree*]	Hamilton et al. (1987)
Feelings	*Worry.* Below is a long list of items people say they worry about. Please read each of the statements and fill in how you feel about each one. Please do not go back and change your answers. 1. Nuclear war . . . [A = *not at all worried*, B = *worried a little*, C = *moderately worried*, D = *very worried*]	Goldenring & Doctor (1986)
	*Physical impacts.* Hearing people talk about all the destruction that could occur if a nuclear bomb were dropped gives me a knot in my stomach. [1 = *never*, 2 = *hardly ever*, 3 = *sometimes*, 4 = *often*, 5 = *always*]	Christie & Hanley (1994)
Action	How much time do you spend thinking/talking/reading/watching programs about the threat of nuclear war? [1 = *never*, 5 = *always*]	Hamilton et al. (1987)
	Each of the questions below describes an activity that relates to the issue of nuclear weapons. Read each item and then indicate whether and how many times you performed that activity during the last 4 years. 1. Turned a conversation to the subject of nuclear weapons so you could present an anti-nuclear weapons view. . . . 12. Signed a pro-nuclear weapons petition. [3 = *three or more times*, 2 = *two times*, 1 = *one time*, 0 = *never*]	Werner & Roy (1985)
Knowledge	Some countries around the world have built nuclear weapons. Of the countries listed below, which, if any, do you believe to be currently in possession of nuclear weapons? Please select all that apply.	Fialho (2021)
	As far as you know, which of the following, if any, are the likely effects of a nuclear weapon explosion: genetic mutation, fire, erosion, hurricanes, loss of fertility, or none of these? Please select all that apply.	Fialho (2021)

Note: Example items have been edited slightly for brevity and style. Answer options are shown in brackets. Scale references are provided in Supplementary Information S7 in the Supplemental Material.

A total of 22 (8%) articles drew on validated scales for exploring different beliefs about nuclear weapons (Table S6). Those asked about perceived nuclear war likelihood generally (S.T. Fiske, 1987; see S.T. Fiske et al., 1983; Rounds & Erdahl, 1988) or within the next 10 (Hamilton et al., 1987; Newcomb, 1985, 1986), 20, or 30 (Nelson et al., 1986; Nelson & Slem, 1984) years. Other scales assessed the perceived likelihood of Australia (Jennings & Lawrence, 1986) or the United States being involved in a nuclear war within a participant’s lifetime (Gwartney-Gibbs & Lach, 1991; Zweigenhaft et al., 1986), the general occurrence of a war within one’s lifetime (Chandler, 1991; de Rivera, 1994), or the likelihood of a Russian attack on the United States (Mayton, 1984). One scale measured whether children thought that nuclear war was generally possible (Stillion et al., 1988).

Belief measures also addressed perceived nuclear war impact (S.T. Fiske, 1987; S.T. Fiske et al., 1983) globally (Larsen, 1985; Mayton, 1984) or in the United States (Mayton, 1984). Related measures asked about chances of survival (Hamilton et al., 1987; Newcomb, 1985, 1986; Zweigenhaft et al., 1986), destruction of political and economic systems (see Mayton, 1988; Nelson & Slem, 1984; Nelson et al., 1986), or whether more generally the world (Larsen, 1985) or the United States (Gwartney-Gibbs & Lach, 1991) would survive a nuclear war. Addressing the perceived aftermath of nuclear war more broadly included food availability, medical help, political structures, and shelter effectiveness (Zweigenhaft et al., 1986).

Some scales also measured broader beliefs about how (Hamilton et al., 1987) or whether humans can deal with nuclear weapons or beliefs about the overall usefulness of nuclear weapons for preventing war (Jennings & Lawrence, 1986). This also included beliefs about the perceived success of different political strategies regarding security (Larsen, 1985), disarmament (Hamilton et al., 1987; Larsen, 1985), defense (Mayton, 1988), governmental cooperation for ending a nuclear conflict, or a nuclear freeze (Stillion et al., 1988; Zweigenhaft et al., 1986). Other scales assessed beliefs about deterrence, whether nuclear weapon use in Hiroshima was useful (Horvath, 1996a), or whether oneself, powerful others, or chance shape nuclear policies (Jennings & Lawrence, 1986; Rounds & Erdahl, 1988).

A total of eight (3%) articles drew on validated scales for exploring feelings (Table S6). Some studies also measured these feelings using scales for general clinical anxiety, but primed participants about nuclear war beforehand (e.g., Hamilton et al., 1989).

Chandler’s (1991) scale suggested several dimensions of nuclear anxiety. First, “despair” included hopelessness and powerlessness about the future because of nuclear weapons and war (see also Christie & Hanley, 1994). Relatedly, others measured nuclear anxiety through other students’ (Stillion et al., 1988) or one’s own “worry” about nuclear war (Christie & Hanley, 1994), nuclear weapon use, the wish to survive a nuclear war (Goldenring & Doctor, 1986; Zweigenhaft et al., 1986), or being “frightened” about nuclear weapons (Newcomb, 1985, 1986).

Second, anxiety included perceived “urgency” of acting on a nuclear threat (Chandler, 1991). This included prioritization of, for example, eliminating the possibility of nuclear war, accepting high costs, or forbidding the building of nuclear weapons.

Third, “denial” (Chandler, 1991) items measured overly optimistic views of the human capacity to deal with weapons. Relatedly, Gwartney-Gibbs and Lach (1991) examined psychic numbing by asking respondents whether nuclear war was generally considered a critical issue and whether they try to learn about this issue and whether they discuss or block feelings related to it.

Some scales also measured perceived somatic impacts of anxiety, such as feeling nervous, increased heart rate, a knot in the stomach (Christie & Hanley, 1994; Stillion et al., 1988), disturbed sleep, crying, or nightmares (Stillion et al., 1988).

A total of 10 (4%) articles drew on validated scales for exploring action (Table S6). One scale assessed people’s self-reported pro- and antinuclear behaviors (Werner and Roy, 1985), including conversations about nuclear weapons (Jennings & Lawrence, 1986), donations to nongovernmental organizations, petition signing, or activist meeting attendance.

Other scales included just single items about behaviors. These assessed the frequency of thinking, talking, or watching programs about nuclear issues (Hamilton et al., 1987; Mayton, 1988) and general policy support of a nuclear freeze or of an increase in U.S. nuclear weapons’ capabilities (Hamilton et al., 1987). Beer et al. (1992) included measures addressing various decisions related to conflicts between states, including the use of various nuclear weapons.

A total of two (1%) articles drew on validated scales for exploring knowledge (Table S6). Fialho (2021) developed a scale measuring perceived knowledge about nuclear weapon states, the number of weapons worldwide, nuclear weapons programs, and the testing as well as impact of previous weapon use. Relatedly, Zweigenhaft et al. (1986) also addressed physical radiation impacts and false-alarm frequency. Overall, the development of validated scales for different response types suggests some progress in the field. Those scales were not widely used, however.

## Discussion

Psychologists have explored people’s responses to nuclear weapons. Here, we systematically mapped how psychologists and scientists from related fields studied public responses to nuclear weapons since the Cold War ended and since several major reviews appeared (S.T. Fiske, 1987; T.W. Graham, 1988; Schatz & S.T. Fiske, 1992). Amid the globally “extremely dangerous” nuclear situation (Bulletin of the Atomic Scientists, n.d.), we sought to assess the current state of empirical research on psychological responses to nuclear weapons. We report four key findings.

First, publication patterns, including the overall number of articles published since the last review (*N* = 256; S.T. Fiske, 1987) may suggest that such research is still a niche research area, contrasting experts’ concerns about nuclear weapons. In comparison, many more articles appeared on psychological responses to other global risks. It was fivefold for the psychology of emotions and climate only within 15 years (Momenpour & Choobchian, 2025) and almost tenfold for psychological research on COVID-19 within 2 years only (Dong et al., 2022). Moreover, most articles per year were published around the end of the Cold War and were often authored by psychologists. This was followed by 2 decades of relative quietude across fields (Fig. 2). This pattern contrasts with the strong interest in public responses to nuclear weapons within and beyond psychology until the end of the Cold War (S.T. Fiske, 1987; Morawski & Goldstein, 1985). Perhaps, post-Cold War global disarmament efforts (Kristensen et al., 2025; Wisotzki & Kühn, 2021) made research on nuclear weapons seem less urgent. Moreover, although these weapons never vanished, this pattern of results is consistent with nuclear weapons possibly also being less salient to psychologist cohorts born around or after the end of the Cold War (Rattinger, 1987).

Since 2020, the number of articles then increased again, mostly within political science and international relations. This increase may reflect the second wave of empirical studies and surveys on the nuclear weapon nonuse norms, including the nuclear taboo (Smetana & Wunderlich, 2021; Tannenwald, 2007). They may also reflect research on attitudes toward nuclear weapons policies in, for example, Europe (e.g., Egeland & Pelopidas, 2021; Smetana & Onderco, 2022), the United States (Baron & Herzog, 2020), or Asia (e.g., Baron et al., 2020; Son & Park, 2022), or heightened expert concern about nuclear weapons (Bulletin of the Atomic Scientists, n.d.). We did not find many articles during our review that specifically addressed how the Russian-Ukrainian war that began in February 2022 may have reshaped public responses to nuclear weapons (but see Riad et al., 2023). Early data from Germany and the Netherlands were offered by Onderco et al. (2023). An active conflict involving nuclear weapon use may shift public responses to nuclear weapons. Understanding shifts will require extensive and sustained research to understand the validity, replicability, and generalizability of initial findings across samples, including from countries that are affected by war (Sinovets & Vicente, 2023). Overall, the U-shaped pattern contrasts the often linear, or even exponential, rise of publications on similar topics (e.g., Zheng et al., 2025).

Second, an analysis of the coauthor network and number of publications per author showed a low network density: Many authors followed a solitary publication strategy, publishing alone, in dyads, or in small groups (Figs. 3a and 3b). Few authors collaborated across groups, acting as potential brokers (Engler et al., 2024). Coauthor network analyses emerging in other fields responding to global risks were denser (e.g., on AI in healthcare or psychology, see Abdulsalam et al. 2025; on climate change and health, see Zheng et al., 2025) or showed single larger author clusters with more publications per author (on degrowth, see Engler et al., 2024). Articles were also scattered across journals from various fields.

The structure of the coauthor network and the dispersed journal landscape suggest several possible explanations. On one hand, researchers from different fields may investigate related questions on responses to nuclear weapons, reflecting that public responses to nuclear weapons are relevant to different fields. On the other hand, authors from different fields may not necessarily share knowledge or research questions that could foster cross-disciplinary cohesion.

Third, two major thematic clusters emerged through an analysis of cocitations. The first cluster reflected psychological research on beliefs, feelings, and actions, often published toward the end of the Cold War. The second cluster reflected more recent research, often conducted within political science and international relations, focusing on public approval of nuclear weapon use and policy support. These clusters thus align with the publication pattern shown in Figure 2. Moreover, we identified a broad range of theoretical backgrounds, mostly from psychology and political science. Some authors referred to well-established theories for explaining beliefs, such as the psychometric paradigm, the social amplification of risk framework, or heuristics. Only few referred to well-established theories for explaining feelings, such as Lazarus’s stress and coping theory, or explained action through the well-established theory of planned behavior.

Fourth, increasing sample sizes may indicate improved research practices regarding more easily available, highly powered samples (Lakens, 2022) and overall increasing sample sizes in psychological studies (Bakker et al., 2025), mostly online (Lukács et al., 2023). In addition, samples were mostly drawn from populations of Westernized countries, with a majority coming from the United States and the United Kingdom; fewer from Australia, Europe, South Korea, and Japan (Fig. S3b); and a handful from non-Westernized countries, such as Russia, China, Brazil, or Morocco. This aligns with behavioral science more broadly, in which samples are often from Western, educated, industrialized, rich, and democratic (WEIRD) countries, such as the United States and United Kingdom (Henrich et al., 2010).

Some articles reported on studies with more specific, perhaps more vulnerable, subgroups, such as children and adolescents, older adults, or (veteran) workers from nuclear weapons production sites. Strikingly few articles reported research involving people affected by nuclear weapon detonations before and after Hiroshima and Nagasaki had been bombed. Those were surveys and interviews with participants from Semipalatinsk, Kazakhstan (Kawano et al., 2006; Kawano & Ohtaki, 2006; Matsuo et al., 2006; Purvis-Roberts et al., 2007). We did not identify any studies that focused on other sites, such as in the Pacific (Li, 2024), Algeria, Australia, or even New Mexico and Nevada in the United States. A similar pattern emerged regarding research including people impacted by radiation and pollution from nuclear weapon production.

Moreover, validity increases when findings converge across different methodological approaches but is limited when researchers all develop their own theories and measures (Mischel, 2008). We identified many validated scales for assessing beliefs, such as nuclear war likelihood and impacts, or effectiveness of different nuclear policy strategies, such as deterrence, governmental cooperation, or a nuclear freeze. Some scales measured dimensions of nuclear anxiety, namely despair, urgency, denial, and self-reported somatic reactions. A more recent nuclear anxiety scale was published after we had conducted the article search (Ćudina et al., 2024). We found five scales that assessed self-reported behaviors, such as the frequency of thinking, talking, or watching programs about nuclear issues as well as political action, and two scales that assessed self-reported knowledge (Table S6).

These scales indicate some progress in the research field. They could serve as a toolbox for future research on responses to nuclear weapons: Validated scales improve on diverse ad hoc measures that impair comparisons of results and thus research progress (S.T. Fiske, 1987; Mischel, 2008). At the same time, such scales use self-reports. Self-reports come with limited validity because of reporting biases and individuals’ limited memory capacity; responses are often, overall, inconsequential, even within one study. Variable relationships in studies using these scales may thus be overestimated (Lange, 2022). Still, this set of scales represents a useful start for future studies. Given that most of these scales date back to the end of the Cold War, they may need to be validated with more recent samples (Elson et al., 2023). Validation should be in line with research guidelines for authors, editors, and reviewers that address how excessive variability in measures can hinder comparisons between studies (Elson et al., 2023). Future studies may also complement these scales with observational data, for example, by developing research paradigms for studying behaviors with higher external validity (Lange, 2022). This may be particularly relevant for decisions about nuclear weapon use that were studied, for example, with short, abstract survey vignettes (Sagan & Valentino, 2017; Slovic et al., 2020). Such vignettes may echo the actual decisions political leaders would be forced to make in a nuclear crisis, but probably only to some extent: Experimental methods may favor specific response types (Lejarraga & Hertwig, 2021).

Future research on people’s responses to nuclear weapons could start by addressing the gaps identified above. This could include the use of understudied geographic and demographic samples that are particularly vulnerable to nuclear weapon impacts and addressing how these samples were or would be affected. For example, it is important to examine not only environmental impacts of nuclear weapons (National Academy of Sciences, Engineering, and Medicine, 2025) but also what different people know about these impacts. The science of science communication (Bruine de Bruin & Bostrom, 2013) helps finding out how what people need and want to know about such impacts. This approach has been successfully used for understanding public responses to other risks, related to health (McDowell et al., 2016), terrorism (Fischhoff, 2011), and climate change (Amelung et al., 2016; Bruine de Bruin et al., 2021), but not yet regarding nuclear weapons. Other examples include up-to-date studies about action as well as policy support regarding nuclear weapons, for example, of the TPNW. Existing studies (e.g., Onderco et al., 2021), however, cannot be generalized to public audiences from other non-TPNW member states. Moreover, public support shapes nuclear policy. It is thus important to provide a comprehensive picture of policy support both generally as well as within population subgroups, such as post-Cold War generations. Younger people were studied thoroughly toward the end of Cold War. We argue for reviving such research because young people will have to live with the impacts of nuclear weapons much longer. Research about climate change, for example, already reflects a focus on such subgroups (Neas et al., 2022). Other ways of addressing the issue of WEIRD samples are large-scale research cooperations across a multitude of countries, such as on climate-mitigation action on individual levels (Vlasceanu et al., 2024), perceptions of extreme weather (Cologna et al., 2025), or trust in science (Mede et al., 2025). Journal editors can provide incentives and explicit targets for more diverse samples (Global Environmental Psychology, 2025). Authors also need to carefully describe sample demographics, including comparisons to the overall population (Rad et al., 2018).

Second, because many articles focused on nuclear weapons and war, it is important to address people’s responses to other stages of nuclear weapons programs (Greenberg et al., 2007a, 2007b), such as nuclear weapon production processes (starting with uranium mining, weapon design, and testing), weapons program sustainment and modernization, the dismantling and disposition of weapons and materials, and the elimination of nuclear weapon programs. This would better reflect what was recently referred to as “slow daily violence” coming with the mere existence of nuclear weapons, even before they are used in war (UN, 2025a).

Third, future research may engage more with more recent psychological theory that attempts to explain responses to nuclear weapons. Examples for studying beliefs and attitudes are social sampling theory (Brown et al., 2022) or mechanisms such as the description-experience gap (Lejarraga & Hertwig, 2021). Insights about how to protect people against misinformation (Ecker et al., 2022) may also help clarify why people may put weight on some instances of information about nuclear weapons and disregard others. Established models from social psychology (e.g., S.T. Fiske, 2018) may help predict against whom people are willing to use nuclear weapons or when they engage in collective action (Agostini & Zomeren, 2021; Fritsche et al., 2018).

Last, future psychological reviews should address clinical mental-health outcomes of nuclear weapons during all stages of nuclear weapons programs. This would allow the public costs of nuclear weapons programs to be better understood, even before nuclear weapon use. This should include second order impacts from, for example, nuclear winter (National Academy of Sciences, Engineering, and Medicine, 2025) onto clinical mental-health outcomes.

To advance research on public responses to nuclear weapons during this period of heightened global risk, we recommend fostering greater interdisciplinary collaboration. Establishing stronger networks among psychologists, political scientists, and international relations scholars can facilitate knowledge sharing and promote more cohesive, interdisciplinary research clusters. One path to create a forum for such work may be to launch a new journal. This could serve as a focal point for interdisciplinary research on public responses to nuclear weapons, fostering greater coherence, collaboration, and visibility within this diverse field. It may also help establish a clearer identity for the domain and attract sustained scholarly attention. We also recommend broadening the scope of research to include non-Western populations by drawing on non-English publications that describe responses from, for example, Russia and Asia. Expanding studies to include regions directly affected by nuclear weapon use, such as in the Pacific or Central Asia, will provide a more comprehensive and differentiated understanding.

We recommend diversifying methodological approaches. Combining self-reports with preregistered behavioral experiments, longitudinal studies, and real-world observational data will enhance the validity and relevance of findings, particularly on complex and high-stakes nuclear decision-making processes. Interdisciplinary funding is essential to sustain research efforts, encourage innovative methodologies, and ensure that psychological insights contribute to national and international policy discussions to reduce nuclear risk through arms control and disarmament.

Future reviews may focus on responses from raw poll data (T.W. Graham, 1988) or gray literature reporting various responses to nuclear threat, such as people’s policy support (T.W. Graham, 1988). Future reviews may also include non-English publications that describe data from, for example, Russia and Asia—the ones described here were all English peer-reviewed articles.

## Conclusion

Empirical evidence on public responses to nuclear weapons, across fields, often addressed related questions but substantially varied in measures. Such research matters given globally increasing nuclear threat. We thus call for stronger cooperation across fields for producing urgently needed knowledge about responses to nuclear weapons (S.T. Fiske, 1987). Psychologists have much to contribute by developing valid and robust measurement methods and by contributing insights about the mechanisms driving responses to nuclear weapons. Our work aims to develop the evidence needed for better understanding and ultimately reducing this existential risk.

## Supplemental Material

sj-docx-1-pps-10.1177_17456916251404895 – Supplemental material for People’s Responses to Nuclear Weapons: Mapping Post-Cold War Research Supplemental material, sj-docx-1-pps-10.1177_17456916251404895 for People’s Responses to Nuclear Weapons: Mapping Post-Cold War Research by Astrid Kause, Helen Fischer, Zia Mian and Susan T. Fiske in Perspectives on Psychological Science

## Transparency

*Action Editor:* Katarzyna Adamczyk

*Editor:* Arturo E. Hernandez
