# Supplementary material for: People’s Responses to Nuclear Weapons: Mapping Post-Cold War Research
Source: Perspect Psychol Sci. 2026 Feb 26;21(3):309–32. doi: 10.1177/17456916251404895 (PMC13096629; doi:10.1177/17456916251404895)
Supplement: sj-docx-1-pps-10.1177_17456916251404895 – Supplemental material for People’s Responses to Nuclear Weapons: Mapping Post-Cold War Research [file sj-docx-1-pps-10.1177_17456916251404895.docx]

**People’s Reactions to the Nuclear Weapons’ Threat:**

**Mapping Post-Cold-War Research and Evidence**

– Supplemental Material –

**Table S1**

*Search Strings Used in SCOPUS and Web of Science*

| **Database** | **Full string** |
| --- | --- |
| SCOPUS 01/2024 | TITLE-ABS-KEY ( ( nuclear OR radioactiv* ) W/3 ( war* OR weapon* OR bomb* OR threat* OR confront* OR age OR force* OR taboo* OR strike* OR attack* OR explosion* ) AND ( opinion* OR support* OR think* OR view* OR believ* OR belief* OR estimat* OR perceiv* OR perception* OR trust* OR know* OR attitud* OR feel* OR affect* OR emot* OR concern* OR preference* OR behavior* OR behaviour* OR react* OR accept* OR support* OR choice* OR behav* OR intent* OR respon* OR decision* OR decid* OR engag* OR act* ) AND ( survey* OR study* OR studies OR questionnaire* OR experiment* OR interview* OR examin* OR measure* OR investig* OR poll* ) ) AND PUBYEAR > 1986 AND PUBYEAR < 2025 AND ( LIMIT-TO ( DOCTYPE , "ar" ) ) AND ( LIMIT-TO ( LANGUAGE , "English")) |
| SCOPUS 04/2024 | ( TITLE-ABS-KEY ( ( nuclear OR radioactiv* ) W/3 ( freeze OR race* OR *arm* ) AND ( opinion* OR support* OR think* OR view* OR believ* OR belief* OR estimat* OR perceiv* OR perception* OR trust* OR know* OR attitud* OR feel* OR affect* OR emot* OR concern* OR preference* OR behavior* OR behaviour* OR react* OR accept* OR support* OR choice* OR behav* OR intent* OR respon* OR decision* OR decid* OR engag* OR act* ) AND ( survey* OR study* OR studies OR questionnaire* OR experiment* OR interview* OR examin* OR measure* OR investig* OR poll* ) ) AND PUBYEAR > 1986 AND PUBYEAR < 2025 ) AND ( LIMIT-TO ( DOCTYPE , "ar" ) ) AND ( LIMIT-TO ( LANGUAGE , "English" ) ) |
| Web of Science 01/ 2024 | TS=( (nuclear OR radioactiv*) NEAR/3 (war* OR weapon* OR bomb* OR threat* OR confront* OR age OR force* OR taboo* OR strike* OR attack* OR explosion*) AND (opinion* OR support*OR think* OR view* OR believ* OR belief* OR estimat* OR perceiv* OR perception* OR trust* OR know* OR attitud* OR feel* OR affect* OR emot* OR concern* OR preference* OR behavior* OR behaviour* OR react* OR accept* OR support* OR choice* OR behav* OR intent* OR respon* OR decision* OR decid* OR engag* OR act*) AND (survey* OR study* OR studies OR questionnaire* OR experiment* OR interview* OR examin* OR measure* OR investig* OR poll*))  [Settings in Web of Science set manually similarly reflected a focus on ‘articles’ published after 1986, and in English language] |
| Web of Science 04/2024 | TS=( (nuclear OR radioactiv*) NEAR/3 (freeze OR race* OR *arm*) AND (opinion* OR support*OR think* OR view* OR believ* OR belief* OR estimat* OR perceiv* OR perception* OR trust* OR know* OR attitud* OR feel* OR affect* OR emot* OR concern* OR preference* OR behavior* OR behaviour* OR react* OR accept* OR support* OR choice* OR behav* OR intent* OR respon* OR decision* OR decid* OR engag* OR act*) AND (survey* OR study* OR studies OR questionnaire* OR experiment* OR interview* OR examin* OR measure* OR investig* OR poll*)) |

**Table S2**

Screening Criteria Used During Title and Abstract as well as Full Text-Screening

|  | **Criterion** | **Decision** | | **Screening stage** | |
| --- | --- | --- | --- | --- | --- |
|  |  | **Inclusion** | **Exclusion** | **Title/**  **abstract** | **Full text** |
| Population | Individuals such as members of the general population, policy makers, non-governmental organizations or the third sector; specific population subgroups such as diplomats, health care workers, children, … | x |  | x | x |
| Population | Studies with focus on group-level only, if they specifically address psychological processes (social networks, collective identity, social influence, ...) | x |  | x | x |
| Population | Studies that assess responses not on individual-level, i.e. states, organizations, schools, hospitals, other larger groups |  | x | x | x |
| Population | Experts, if study reports expert assessments about technical aspects of nuclear technology (such as strategic stability, nuclear weapon programs or emerging technology and nuclear security) |  | x | x | x |
| Population | Media analyses assessing views expressed in newspaper articles written by professional journalists and NOT individual traces (do only very indirectly allow making conclusions about individuals’ views) |  | x | x | x |
| Outcome | Perceptions, feelings and actions / behaviors in response to nuclear danger, or knowledge about nuclear danger; assessed through direct (introspective) response or observation | x |  | x | x |
| Outcome | Traces individuals directly leave in media or the like; such as tweets, comments in forums, traces can be directly observed in peoples' socio-cultural environments | x |  | x | x |
| Outcome | Simulation results if model agents represent individuals/ humans and if some type of empirical data and/ or psychological mechanism feeds into simulation | x |  | x | x |
| Outcome | Studies including one item assessing measuring the above outcomes should be included. | x |  | x | x |
| Outcome | Responses to nuclear danger can either be the predictor of a different variable, such as savings decisions or fear of the future, or be predicted with variables such as values, attitudes, age, personality, anxiety scores, political attitude or the like | x |  | x | x |
| Outcome | Disaster/ emergency preparedness of health care practitioners if study explicitly addresses responses to nuclear weapons | x |  | x |  |
| Outcome | Disaster/ emergency preparedness of health care practitioners if focus is on nuclear weapons, or chemical, biological, radiological, nuclear weapons more generally |  | x |  | x |
| Outcome | Disaster/ emergency preparedness on general levels and in response to a wider variety of incidents, including nuclear weapons, but study/ studies do not include items that specifically mention nuclear weapons, attacks or the like |  | x | x | x |
| Outcome | Studies that explicitly assess mental health and health outcomes more generally, including sleep, mental disorder, depression, cognitive functioning or the like |  | x |  | x |
| Outcome | Media analyses assessing views expressed in newspaper articles written by professional journalists and NOT individual traces (do only very indirectly allow making conclusions about individuals’ views) |  | x | x | x |
| General | Focus on any non-civilian use of nuclear technology, including weapons, test sites, terrorism, policies that address nuclear dangers, or public preparedness | x |  | x | x |
| General | Studies reporting empirical data that was collected using an established method from psychology or related fields; could be observational, correlational, experimental; qualitative and quantitative; mixed methods | x |  | x | x |
| General | Reviews of empirical studies that fit into the overall scope of this review | x |  | x | x |
| General | Case study descriptions; analyses of meeting notes, policy documents etc.. Those may summarize views, but leave unclear who said what and why content made its' way into documents |  | x | x | x |
| General | Civilian use of nuclear energy, general perception of nuclear waste where origin of waste is unclear |  | x | x | x |
| General | Study does not specify whether a nuclear emergency is from non-civilian danger, but on radiological disaster more generally, including preparedness for CBRN incidents |  | x | x | x |
| General | Conceptual articles, opinion papers, commentaries |  | x | x | x |
| General | Articles describing tools, frameworks and the like without testing those empirically |  | x | x | x |
| General | Historical articles that are not an explicit review of empirical literature |  | x | x | x |
| General | Articles describing 'some experience' with X, without specifying an established method for empirical data collection and analysis |  | x | x | x |
| General | Case studies that do not report an established method for empirical data collection and analysis |  | x | x | x |

**Table S3**

*Manual Coding Scheme Applied to Included Articles*

| **Category** | **Description & Subcategories** |
| --- | --- |
| **Study Characteristics** | |
| First Author Location | [Country] |
| First Author Department | [Open text field] |
| **Study Background** | |
| Study topic | 1. Describes general study topic. Select from: Nuclear weapons (direct), nuclear (or risk thereof), weapon production sites, nuclear terrorism, nuclear policy, nuclear weapons waste management, emergency preparation/ warnings, deterrence, other, [open text field for ‘other’] 2. Do authors see nuclear weapons as something positive (enhancing national security), neutral, or negative (nuclear weapons need to be abandoned) |
| Theory | 1. Theory cited in introduction. Examples: Theory of planned behavior, deterrence theory, psychometric paradigm, accentuation theory, construal level theory, … [list and open text field] 2. Theory – central references cited [open text field] |
| Mechanism/ Effect | 1. Quote precise mechanism that authors draw on for making either precise predictions or explaining specific (causal) patterns in responses. Examples: Psychic numbing, availability, security prominence, nuclear taboo 2. Mechanism/effect – central reference cited [open text field] |
| Research Question/ Aim | Explicitly mentioned research questions or aims [open text field] |
| Hypotheses | Quote hypotheses [open text field] |
| **Method** | |
| Causality | Indicates whether authors assume to be addressing causal relationships. [Select from: Yes, no, unclear] |
| Method - General | [Select from: Qualitative, quantitative, mixed] |
| Method - Specific | [Select from: Poll, survey, experiment, quasi-experiment, controlled observation, interview, focus group, media data [such as twitter], other traces reflecting peoples’ decisions such as investment decisions or insurance data]; includes open text field for describing experimental design where appropriate |
| Design | Applies to experiments only: Specify experimental design reported [open text field] |
| Preregistration | [Select from: Yes/ No/ Partially] |
| Number of studies in paper | [Select from list with numerical options 1-7] |
| **Sample** | |
| Location | Country |
| Size | Provide N. If several studies are reported, include sum of all N’s [open text field] |
| Type | [Select from: General population, students, elderly, adolescents, children, activists, experts, other; open text field for ‘other’] |
| Population -location | Country code |
| Population – type | [Select from: General population, students, elderly, adolescents, children, activists, experts, other; open text field for ‘other’] |
| **Variables** | |
| Variables addressed | 1. General. [select from: beliefs, feelings, actions, knowledge, other] |
| Predictor | 1. Variable predicting beliefs, feelings, actions, knowledge 2. Description of exact predictor [open text field], including reference |
| Manipulated variable | Only applies to experiments and quasi-experiments. Specify what authors manipulated, such as vividness of information, seeing a film versus not, etc. [open text field] |
| Measured variable | 1. Specific – select from: Political attitudes, attitudes towards weapons, attitudes towards launch, nuclear anxiety, different types of behaviors … 2. Quote [open text field] |
| Standardized measure | Open text field for describing measurement scale authors reported to use |
| Covariates | Any covariates authors report to control for [Select from: Gender, age, education, ethnicity, location, SES, party affiliation, political orientation (left-right), Trump supporter, Efficacy (own, group), Other, open text field for ‘other’] |
| **Findings** |  |
| Main finding | Main study finding (from results or intro of discussion) [open text field] |
| Finding - description | [Select from positive (as predicted), negative (against prediction), neutral, mixed, no hypothesis] |
| Implication | Policy implication of main findings [open text field] |
| Other | [open text field] – any other comment coder has on article |

**Table S4**

*Journals That Included More than One Article, Number of Articles/ Journal, SciMago Journal Rank and Journal Impact Factor (as of April 2025)*

| Journal | Number of articles | SciMago Journal Rank  (if not stated differently: SJR Q1, 2024) | Journal Impact Factor (if not stated differently: JIF 2023) |
| --- | --- | --- | --- |
| Adolescence | 2 | 0,467 (Q1, 2012) | 0,6 (2011) |
| American Journal of Orthopsychiatry | 4 | 1,068 | 2,3 |
| American Psychologist | 2 | 3,2 | 12,3 |
| Australian Journal of Political Science | 2 | 0,476 (Q2, 2024) | 1,2 |
| Australian Psychologist | 2 | 0,816 (Q2, 2024) | 2 |
| Basic and Applied Social Psychology | 2 | 0,947 (Q2, 2024) | 2,5 |
| British Journal of Social Psychology | 5 | 1,665 | 3,2 |
| Bulletin of the Atomic Scientists | 2 | 0,755 | 1,9 |
| Conflict Management and Peace Science | 2 | 1,509 | 1,7 |
| Current Psychology | 2 | 1,024 | 2,5 |
| Death Studies | 2 | 0,969 | 2,1 |
| Environment and Behavior | 2 | 1,483 | 5,2 |
| European Security | 2 | 1,342 | 2,7 |
| Foreign Policy Analysis | 3 | 0,762 | 1,7 |
| Frontiers in Public Health | 2 | 1,027 | 3 |
| Global Policy | 2 | 0,631 | 2,2 |
| Human Ecology Review | 2 | 0,197 (Q3, 2024) | 0,8 |
| International Interactions | 3 | 0,56 | 1,5 |
| International Journal of Communication | 2 | 0,653 | 1,9 |
| International Journal of Environmental Research and Public Health | 2 | 0,919 (Q2, 2024) | 4,6 (2021) |
| International Journal of Public Opinion Research | 2 | 0,794 | 1,9 |
| International Security | 3 | 2,371 | 4,8 |
| International Studies Quarterly | 4 | 1,518 | 2,4 |
| Journal for Peace and Nuclear Disarmament | 2 | 0,261 (Q2, 2024) | 0,5 |
| Journal of Adolescence | 4 | 1,506 | 3,0 |
| Journal of Applied Social Psychology | 13 | 1,018 (Q2, 2024) | 2,2 |
| Journal of Conflict Resolution | 7 | 2,138 | 2,2 |
| Journal of Global Security Studies | 4 | 1,062 | 1,7 |
| Journal of Peace Research | 9 | 1,941 | 3,4 |
| Journal of Radiation Research | 3 | 0,603 (Q2, 2024) | 1,9 |
| Journal of Social Behavior and Personality | 3 | 0,282 (2004) | 0,283 (2001) |
| Journal of Social Issues | 2 | 1,746 | 4 |
| Medicine, Conflict and Survival (originally published as “Medicine and War”) | 4 | 0,371 (Q3, 2024) | NA |
| National Bureau of Economic Research (NBER Working Paper) | 2 | NA | NA |
| Peace and Conflict: Journal of Peace Psychology | 4 | 0,465 (Q2, 2024) | 0,9 |
| Perceptual and Motor Skills | 2 | 0,606 (Q3, 2024) | 1,4 |
| Personality and Social Psychology Bulletin | 3 | 2,174 | 3,4 |
| Political Psychology | 4 | 2,047 | 4,0 |
| Psychological Reports | 4 | 0,763 (Q2, 2024) | 1,7 |
| Public Opinion Quarterly | 4 | 1,721 | 2,9 |
| Research & Politics | 2 | 1,14 | 2,0 |
| Risk Analysis | 6 | 0,869 | 3,0 |
| Security Studies | 3 | 1,414 | 2,2 |
| Sex Roles | 3 | 1,264 | 3,0 |
| The Journal of Politics | 3 | 3,428 | 3,5 |
| The Journal of Psychology | 2 | 0,929 | - |
| The Journal of Social Psychology | 5 | 0,945 (Q2, 2024) | 1,8 |
| The Nonproliferation Review | 3 | 0,250 (Q3, 2024) | NA |
| The Social Science Journal | 2 | 0,535 (Q2, 2024) | 1,8 |
| The Sociological Quarterly | 2 | 0,568 (Q2, 2024) | 1,2 |

*Note.* Ninety-five journals published but one article. Journal information was retrieved from https://www.scimagojr.com/journalrank.php and https://mjl.clarivate.com/home

**Table S5a**

*Broader Frameworks/ Paradigms Reported in Articles*

| Frameworks / Paradigms | Example references |
| --- | --- |
| Deterrence Theory | Avey (2021); Axelrod & Newton (2021); Beer et al., (1992); Ko (2019); Pauly (2018); Simon (2004); Smetana, Vranka & Rosendorf (2023); Son & Yim (2021); Sussman & Steel (1991) |
| Rationality/ Rational Choice | Clary, Lalwani & Siddiqui (2021); Davidson & Newman (1990); Hetsroni, Reizer & Ben Zion (2017); Kramer (1989); Plous (1993; 1987); Quek (2016); Ripberger, Rabovsky & Herron (2011); Russett et al. (1993); Russett & Slemrod (1992); Slemrod (1988); Williams, Brown & Greenberg (1999) |
| Authoritarianism | Clark, Trahair & Gretz (1989); Doty et al. (1997); Rathbun & Stein (2020); Rigby, Metzer & Dietz (1990); Van Uzendoorn (1990) |
| Social Movement | Edwards & Oskamp (1992); Marullo (1988); Mix & Shriver (2009); Tygart (1987) |
| Social Amplification of Risk Framework | Flynn, Peters & Slovic (1998); Lytle & Karl (2020); Mix & Shriver (2009); Williams, Brown & Greenberg (1999) |

**Table S5b**

*Theories Reported in Articles*

| Theory | Example references |
| --- | --- |
| Social Identity / Social Norms | Benford (1993a, 1993b); Cheng (2023); der-Karabetian (1992); Fair, Kaltenthaler & Miller (2013); Gulevich & Osin (2023); Herzog & Baron (2017); Heskin & Power (1994); Horvath (1996); Machida (2018); Machida (2022); Matsumura, Tago & Grieco (2023); Pomeroy (2024); Press, Sagan & Valentino (2013); Reicher & Lewine (1994); Rigby, Metzer & Dietz (1990); Saleh & Gandy, (2015); Trost, Cialdini & Maass (1989); Wohl, Porat & Halperin (2015) |
| Values | Boehnke & Schwartz (1997); Cho (2016); Dehghani et al. (2010); Namkung (2010); Hamilton (1989); Heskin (1994); Horvath (1996); Kristiansen (1990); Mayton (1992a, b); Mayton & Furnham, 1994; Ripberger, Rabovski & Herron (2011); Sussman & Steel (1991) |
| Moral Foundations | Pomeroy & Rathbun (2023); Smetana & Vranka (2023); Dill, Sagan & Valentino (2022); Horschig (2022); Langdon-Koch & Wells (2021); Smetana & Vranka (2021); Rathbun & Stein (2020), van Ujzendoorn (1990) |
| Stress and Coping Theory | Boehnke & Wong (2011); Boehnke & Schwartz (1997); Hamilton (1989, 1987); Horvath (1996); McKenzie‐Mohr, Dyal & McLoughlin (1992); McKenzie‐Mohr & Dyal (1991); Savolainen (2023) |
| Psychometric Paradigm | Bacci, Fabbricatore & Iannario (2023); Cutter (1992); Flynn et al. (1998); Greenberg et al. (2007a, 2007b); Peters, Burraston & Mertz, (2004); Xie, Wang & Xu (2003) |
| Terror Management Theory | Boscarino et al. (2006); Hirschberger, Pyszczynski & Ein Dor (2009); Hirschberger, Pyszczynski & Ein Dor (2015); Horschig (2022) |
| Theory of Planned Behavior; Theory of Reasoned Action | TPB: Hetsroni, Reizer & Ben Zion (2017); Fox-Cardamone, Hinkle & Hogue (2000); Horvath (1996); McClenney & Neiss (1989); Halford et al. (1988)  TRA: Horvath (1996); Axelrod & Newton (1991); McKenzie‐Mohr & Dyal (1991) |
| Protection Motivation Theory | Axelrod & Newton (1991); Horvath (1996); McKenzie‐Mohr & Dyal (1991) |
| Nine Nuclear Orientations | Hamilton et al. (1987); Hamilton et al. (1989); Russo & Lyon (1990) |

**Table S5c**

*Specific Mechanisms Reported in Articles*

| Mechanism | References |
| --- | --- |
| Gender Gap | Bae & Lee (2020); Clements & Thompson (2022); Cutter (1992); Gwartney-Gibbs & Lach (1991); Heskin & Power (1994); Lamare (1989); Lee Fox & Schofield (1989); Maleta (2018); Rabow, Hernandez & Newcomb (1990); Silverman & Kumka (1987); Slee & Cross (1989); Son & Park (2023); Sussman & Steel (1991); Wilkins & Lewis (1993); Xie, Wang & Xu (2003) |
| Elite-public Gap/ Elite Cues | Allison, Herzog & Ko (2022); Bowen & Goldfien (2021); Clements & Thompson (2022); Herron & Jenkins-Smith (2002); Herzog, Baron & Davis-Gibbons (2022); Lee (2023); Smetana & Onderco (2022); Son & Park (2023) |
| Numbing | Christie & Hanley (1994); DeMuth & Melnick (1998); Gwartney-Gibs & Lach (1991); Kaplan (1988); Skovholt et al. (1988); Slovic et al (2020); Smith (1988); Valente (1988) |
| Heuristics | Chibnall & Wiener (1988); Peffley & Hurwitz (1992); Peters, Burraston & Mertz (2004); Plous (1989); Xie, Wang & Xu (2003) |
| Locus of Control | Herron & Jenkins-Smith (2002); Rounds & Erdahl (1988); Rudoy, Reznikoff & Geisinger (1987); Stewart (1988) |
| Generation Gap | Herron & Jenkins-Smith (2002); Rattinger (1987); Taylor et al. (2011) |
| NIMBY | Baron & Herzog (2020); Greenberg et al. (2007); Halfacre, Matheny & Rosenbaum (2000) |

**Table S5d**

*Policy Norms or Concepts Reported in Articles*

| Norm/ Concept | References |
| --- | --- |
| Nuclear Taboo | Allison, Herzog & Ko (2022); Avey (2021); Baron & Herzog (2020); Blair, Chu & Schwartz (2022); Bowen Goldfien & Graham (2021); Carpenter & Montgomery (2020); Cheng et al. (2023); Clary, Lalwani & Siddiqui (2021); Dill, Sagan & Valentino (2022); Horschig (2022); Langdon-Koch 2024; Langdon-Koch (2021); Machida (2014); Machida (2018); Onderco, Smetana & Etienne (2023); Onderco et al. (2021); Pauly (2018); Press, Sagan & Valentino (2013); Rathbun & Stein (2020); Reddie & Goldblum (2023); Rosendorf, Smetana & Vranka (2021); Sagan & Valentino (2017); Smetana & Onderco (2021); Smetana, Onderco & Etienne (2021); Smetana, Vranka & Rosendorf (2023); Smetana & Vranka (2023); Sukin (2020) |
| Hawkish/ Dovish Motives | Casler, Ribar & Dohi-Malo (2023); Clark, Trahair & Gretz, (1989); Clary, Lalwani & Siddiqui (2021); Haworth, Sagan & Valentino (2019); Pomeroy & Rathbun (2024); Russett (1991) |
| Retribution/ Retributive Justice/ Retaliation | Allison, Herzog & Ko (2022); Dill, Sagan & Valentino (2022); Langdon-Koch (2024); Langdon-Koch & Wells (2021); Pomeroy (2024); Smetana, Vranka & Rosendorf (2023) |
| Security (Prominence) | Lee (2023); Son & Yim (2023); Egeland & Pelopidas (2021); Slovic et al. (2020) |
| Rally Around the Flag | Clary, Lalwani & Siddiqui (2021); Lamare (1987); Lamare (1991); Russett (1991) |
| Civilian / Non-combatant Immunity Norm | Carpenter & Montgomery (2020); Dill, Sagan & Valentino (2022); Sagan & Valentino (2017) |
| Nuclear ‘Freeze’ | Chibnall & Wiener (1988); Cutter et al. (1987); Hogan & Smith (1991); Ripberger, Rabovsky & Herron (2011) |

*Note.* Tables only list theoretical backgrounds that were identified more than twice. Referenced articles and the full range of theoretical backgrounds extracted are provided at <https://osf.io/jz5xc/>.

**Table S6**

*Validated Scales Cited in Included Articles*

| **Response type** | | | | **Scale** | **Reference** | **Example Articles Citing Scale** |
| --- | --- | --- | --- | --- | --- | --- |
| BELIEF | FEEL | ACT | KNOW |  |  |  |
|  |  | X |  | International Peace and Conflict Database (COBDAB) | Beer et al. (1992) | Beer et al. (1992) |
| X | X |  |  | Nuclear War Anxiety (NWA) | Chandler (1991) | Chandler (1991); Prazeres et al. (2023); Riad et al. (2023) |
| X | X |  |  | Survey of Feelings about the Threat of Nuclear War (SFTNW) | deRivera (1994) | McCarthy (1988) |
| X |  |  |  | Nuclear Locus of Control (NLOC) | Rounds & Erdahl (1988) | Rounds & Erdahl (1988) |
|  |  |  | X | Public Knowledge on Nuclear Weapons (PKNW) | Fialho (2021) | Fialho (2021) |
| X |  | X |  | Nuclear War Attitude Survey (NWAS II) | French & van Hoorn (1986) | Van Hoorn et al. (1989)  Van Hoorn & French (1989) |
|  | X |  |  | Worry about Nuclear War (WAN) | Goldenring & Doctor (1985) | Boehnke & Schwartz (1997); Gillies (1989); Jensen (1988) |
| X |  |  |  | Inventory of Nuclear War Attitudes (INWA) | Grueneich et al. (1983) | Van IJzendoorn (1990) |
| X | X |  |  | Nuclear War Attitudes Index (NWAI) | Gwartney-Gibs & Lach (1991) | Gwartney-Gibs & Lach (1991) |
| X |  | X |  | Nuclear Threat Questionnaire (NTQ) | Hamilton et al. (1987) | Horvath, (1996b); Hamilton, (1989) |
| X |  |  |  | Nuclear Orientation Questionnaire (NOQ) | Hamilton et al. (1987) | Hamilton (1989) |
| X |  |  |  | Nuclear Attitudes Questionnaire (NAQ) | Hamilton et al. (1986); see Mayton (1988) | Mayton (1988) |
| X | X |  |  | Nuclear Anxiety Inventory (NAI) | Hanley (1985); Christie & Hanley (1994) | Christie & Hanley (1994) |
| X |  |  |  | Pro-/Antinuclear Thoughts Questionnaire (PATQ) | Horvath (1996) | Horvath (1996a, b); Hamilton (1989) |
| X | X | X |  | Nuclear Attitudes Questionnaire (NARQ) | Jennings & Lawrence (1986) | Peterson et al. (1990) |
|  |  |  |  | Social Opinions and Perceptions Survey Assessing Nuclear Attitude (SOP) | Kristiansen & Matheson (1990) | Kristiansen & Matheson (1990) |
| X |  |  |  | Attitudes Towards Nuclear Disarmament (AND)* | Larsen (1985) | Gunn & Horvath (1987); Murphy & Polyson (1991) |
|  |  |  |  | Perceptions about nuclear freeze (PANF) | McClennley & Neiss (1989) | Mayton & Sangster (1992) |
| X |  |  |  | Modified World Affairs Questionnaire (MWAQ) | Mayton (1988) | Mayton (1992) |
| X |  |  |  | Spontaneous Concern About the Nuclear Threat Scale (SCANTS) | Mayton (1988) | Mayton (1992) |
| X |  |  |  | Nuclear Attitudes Questionnaire (NAQ)* | Newcomb (1986) | Rabow et al. (1990); Riad et al. (2023); Newcomb et al. (1992) |
| X |  |  |  | Nuclear Weapons Policies Questionnaire (NWPQ)* | Nelson & Slem (1984), Nelson et al. (1986); see also Mayton (1988) | Mayton (1988) |
| X |  |  |  | Nuclear Likelihood Questionnaire (NLQ) | Rounds & Erdahl (1988) | Rounds & Erdahl, (1988) |
| X |  |  |  | Nuclear Locus of Control Scale (NLCS) | Rounds & Erdahl (1988)  [Erdahl & Rounds (1986b)](https://www.semanticscholar.org/paper/Locus-of-Control-and-Likelihood-of-Nuclear-War%3A-Two-Erdahl-Rounds/fe4e99ed5bf0f36ad2c15e5395cddaf8ffd60ac3) | Rounds & Erdahl (1988) |
|  |  | X |  | Nuclear Intentions Questionnaire (NIQ) | Rounds & Erdahl (1988) | Rounds & Erdahl (1988) |
| X |  |  |  | Nuclear Threat Index (NTI) | Stillion et al. (1988) | Klingman & Goldstein (1994); Klingman et al. (1991) |
|  |  |  |  | Nuclear Coping Strategies (NCS) | Stone & Neale (1984) | Hamilton (1989) |
|  |  | X |  | Nuclear Activism Questionnaire (NAcQ) | Werner & Roy (1985) | Axelrod & Newton (1991); de Rivera et al. (1994); Edwards & Oskamp (1992) |
| X | X |  |  | Nuclear knowledge and anxiety scale (NKNA) | Zweigenhaft et al. (1986) | Columbus (1993) |

*Note*. Scales marked with * were found in Mayton (1988), an article originally not included into the review due to lack of empirical data. We could not retrieve the full article of Grueneich et al. (1983) and Erdahl & Rounds (1986; Nuclear Intentions Questionnaire). Full references for all scales are provided in Supplementary Information S7.

**Supplementary Information S7**

References for Validated Scales that Measured Responses to Nuclear Threat (Table 3; 3^rd^ column)

Beer, F. A., Ringer, J. F., Sinclair, G. P., Healy, A. F., & Bourne, L. E. (1992). Ranking international cooperation and conflict events. *International Interactions, 17*(4), 321-348. <https://doi.org/10.1080/03050629208434787>

Chandler, M. A. (1991). Developing a measure of nuclear war anxiety: A factor analytic study. *Humboldt Journal of Social Relations, 16*(2), 39–63. <http://www.jstor.org/stable/23262715>

Christie, D. J., & Hanley, C. P. (1994). Some psychological effects of nuclear war education on adolescents during Cold War II. *Political Psychology*, *15*(2), 177-199. <https://doi.org/10.2307/3791737>

de Rivera. J. (1984) Facing nuclear weapons. *American Behavioral Science, 27*, 739-756. <https://doi.org/10.1177/000276484027006006>

Fialho, F. M. (2021). Measuring public knowledge on nuclear weapons in the post-cold war: Dimensionality and measurement invariance across eight European countries. *Measurement Instruments for the Social Sciences*, *3*(10). <https://doi.org/10.1186/s42409-021-00028-5>

French, P. L., & Van Hoorn, J. (1986). Half a nation saw nuclear war and nobody blinked? A reassessment of the impact of the day after in terms of a theoretical chain of causality. *International Journal of Mental Health, 15*(1–3), 276–297. <https://doi.org/10.1080/00207411.1986.11449034>

Goldenring, J. M., & Doctor, R. (1986). Teen-age worry about nuclear war: North American and European questionnaire studies. *International Journal of Mental Health, 15*(1-3), 72-92. <https://doi.org/10.1080/00207411.1986.11449021>

Grueneich, R., Weldon, D. A., & Zecker, S. G. (1983). *Construction of an attitude scale for the assessment of concern about nuclear war* [Conference presentation]. 54^th^ Annual Meeting of the Eastern Psychological Association, Philadelphia, United States.

Gwartney-Gibbs, P. A., & Lach, D. H. (1991). Sex differences in attitudes toward nuclear war. *Journal of Peace Research, 28*(2), 161-174. <https://doi.org/10.1177/0022343391028002003>

Hamilton, S.B., Knox, T.A., Keilin, W.G. & Chavez, E.L. (1987). In the eye of the beholder: Accounting for variability in attitudes and cognitive/affective reactions toward the threat of nuclear war. *Journal of Applied Social Psychology, 17*(11), 927-952. <https://doi.org/10.1111/j.1559-1816.1987.tb00299.x>

Hamilton, S. B.. Chavez, E. L., & Keitlin, W. G. (1986). Thoughts of Armageddon: The relationship between nuclear threat attitudes and cognitive/emotional responses*. International Journal of Mental Health,* 15(1-3)*,* 189-207. [https://doi.org/10.1080/00207411.1986.11449028](https://psycnet.apa.org/doi/10.1080/00207411.1986.11449028)

Hanley, C. P. (1985). *The development and validation of the nuclear anxiety inventory: analytic study* [Unpublished master's thesis]. The Ohio State University. (ED 302 488). ERIC. <https://files.eric.ed.gov/fulltext/ED302488.pdf>

Horvath, P. (1996a). Antinuclear and pronuclear empowerment and activism. *Journal of Peace Research*, *33*(2), 137-152. <https://doi.org/10.1177/0022343396033002002>

Jennings, P., & Lawrence, J. (1986). *Students and the nuclear arms race* [Research Report]. Murdoch University Australia. <https://core.ac.uk/download/77132613.pdf>

Kristiansen, C. M., & Matheson, K. (1990). Value Conflict, Value Justification, and Attitudes toward Nuclear Weapons. *The Journal of Social Psychology, 130*(5), 665–675. <https://doi.org/10.1080/00224545.1990.9922959>

Larsen, K. S. (1985). Attitudes toward nuclear disarmament and their correlates. *The Journal of Social Psychology, 125*(1), 17–21. <https://doi.org/10.1080/00224545.1985.9713504>

Mayton, D. M. (1988). Measurement of nuclear war attitudes: Methods and concerns. *Basic and Applied Social Psychology, 9*(4), 241–263. <https://doi.org/10.1207/s15324834basp0904_1>

Mayton, D. M. (1988). Psychometric characteristics of the modified world affairs questionnaire. *Psychology: A Journal of Human Behavior, 25*(2), 50–58. <https://eric.ed.gov/?id=EJ386808>

McClenney, L., & Neiss, R. (1989). Psychological responses to the threat of nuclear war. *Journal of Applied Social Psychology, 19*(15), 1239–1267. <https://doi.org/10.1111/j.1559-1816.1989.tb01249.x>

Nelson, L. L., & Slem, C. M. (1984). *Attitudes about arms control and effects of “The Day After."* [Conference presentation]. APA 1984 Convention, Toronto, Ontario, Canada. (ED 257 699). ERIC. <https://files.eric.ed.gov/fulltext/ED257699.pdf>

Nelson, L., Slem, C., & Perner, L. (1986, July). Effects of classroom instruction about the

nuclear arms race. Paper presented at the Annual Meeting of the International Society of Political Psychology, Amsterdam, Netherlands.

Newcomb M. D. (1986). Nuclear attitudes and reactions: Associations with depression, drug use, and quality of life. *Journal of Personality and Social Psychology, 50*(5), 906–920. <https://doi.org/10.1037//0022-3514.50.5.906>

Rounds, J. B., & Erdahl, P. (1988). Nuclear locus of control scales: Information on development, reliability, and validity. *Educational and Psychological Measurement*, *48*(2), 387-395. <https://doi.org/10.1177/0013164488482011>

Stillion, J. M., Goodrow, H., Klingman, A., Loughlin, M., Morgan, J. D., Sandsberg, S., Walton, M., & Warren, W. G. (1988). Dimensions of the shadow: Children of six nations respond to the nuclear threat. *Death Studies*, *12*(3), 227-251. <https://doi.org/10.1080/07481188808252239>

Stone, A. A, & Neale, J. M. (1984). New measure of daily coping: Development and preliminary results. *Journal of Personality and Social Psychology, 46*, 892-906. <https://doi.org/10.1037/0022-3514.46.4.892>

https://documents.un.org/doc/undoc/gen/n24/427/04/pdf/n2442704.pdf

Werner, P. D., & Roy, P. J. (1985). Measuring activism regarding the nuclear arms race. *Journal of Personality Assessment, 49*(2), 181–186. https://doi.org/10.1207/s15327752jpa4902_15

Zweigenhaft, R. L., Jennings, P., Rubinstein, S. C., & van Hoorn, J. (1986). Nuclear knowledge and nuclear anxiety: a cross-cultural investigation. *The Journal of Social Psychology, 126*(4), 473–484. <https://doi.org/10.1080/00224545.1986.9713615>

**Figure S1**

*Methods and Sample Sizes Reported, Over Time*

**

*Note.* Dots show sample sizes (y-axis) reported in N = 256 articles, over time (x-axis), with black dots representing articles reporting on “mixed method” studies; grey triangles on “qualitative” and light gray squares articles reporting on “quantitative” studies. Linear regressions were fitted to publication year and sample size, for each of the three subgroups “mixed,; qualitative,“ and “quantitative“ methods. One article was excluded from this figure for display purpose, due to its exceptionally large sample size (Cutter et al., 1987; N = 284 012).

Quantitative methods ranged from surveys (N = 140; 55%), experiments (N = 64; 25%), and quasi-experiments (N = 11; 4%), to cross-sectional (N = 5; 2%) and longitudinal polls (N = 15; 6%). Qualitative methods included interviews (N = 28; 11%) and focus groups (N = 4; 2%). Only a few articles reported more than one method (N = 26; 10%). Few described a mixed-method approach (N = 16; 6%).

**Figure S2**

*Percentage of Articles, Published by Authors from Different Fields*

*Note.* We extracted authors’ affiliations, as indicated on article PDFs or journal websites. ‘Field’ was coded based on authors’ affiliations.

**Figure S4**

*Frequency of Authors and Samples, per Country*

*Note.* Figure S4A: More than half, namely N = 148 articles (58%) were published by authors located in the US, followed by N = 17 from authors located in the UK (7%) and N = 16 (6%) in Australia. All other authors (N = 86; 33%) were based in various countries, including Japan, Israel, South Korea and the Czech Republic. Figure S4B: A similar pattern was observed for samples reported.
